# Acute effects of negative heel shoes on perceived pain and knee biomechanical characteristics of runners with patellofemoral pain

**DOI:** 10.1002/jfa2.12001

**Published:** 2024-03-29

**Authors:** Yu Gu, Zhiyi Zheng, Quanshou Zeng, Chen Yang, Yu Song, Xianglin Wan

**Affiliations:** ^1^ Department of Sports Biomechanics Beijing Sport University Beijing China; ^2^ ANTA Sports Science Laboratory ANTA (China) Co., Ltd. Xiamen China; ^3^ School of Sports and Health Nanjing Sport Institute Nanjing China; ^4^ Department of Health, Sport, & Exercise Sciences University of Kansas Lawrence Kansas USA

**Keywords:** heel‐to‐toe drop, knee injuries, patellofemoral joint, running, shoes

## Abstract

**Background:**

To determine the effects of negative heel shoes on perceived pain and knee biomechanical characteristics of runners with patellofemoral pain (PFP) during running.

**Methods:**

Sixteen runners with PFP ran in negative (−11 mm drops) and positive (5 mm drops) heel shoes while visual analog scale (VAS) scores, retroreflective markers, and ground reaction force were acquired by applying a 10‐cm VAS, infrared motion capture system, and a three‐dimensional force plate. Knee moment, patellofemoral joint stress (PFJS), and other biomechanical parameters during the stance phase were calculated based on inverse dynamics and a biomechanical model of the patellofemoral joint.

**Results:**

The foot inclination angle, peak PFJS during the stance phase, patellofemoral joint reaction force, knee extension moment, and quadriceps force at the time of peak PFJS of runners with PFP in negative heel shoes were lower than that in positive heel shoes, no significant difference was found in VAS scores, knee flexion angle, patellofemoral contact area, and quadriceps moment arm at the time of peak PFJS.

**Conclusions:**

Compared to positive heel shoes, running in negative heel shoes decreases peak PFJS in runners with PFP, which may decrease patellofemoral joint loading, thus reducing the possibility of further development of PFP.

**Trail Registration:**

Sports Science Experiment Ethics Committee of Beijing Sport University. 2023095H, April 18, 2023 (prospectively registered).

## Graphical abstract

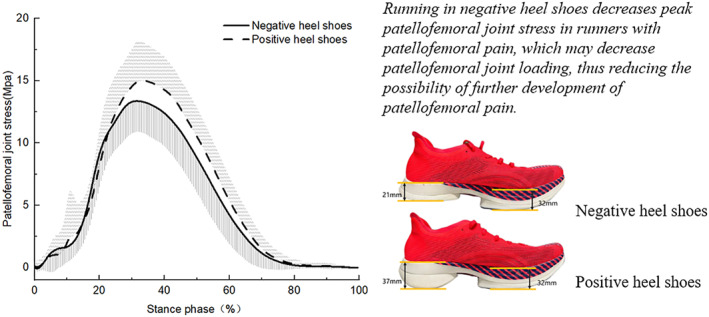

## 1 BACKGROUND

Patellofemoral pain (PFP), commonly known as runner's knee, is one of the most common running injuries, with a prevalence of 16.7% in the running population [1]. PFP is characterized by retro‐patellar or peri‐patellar pain during activities such as squatting, ascending and descending stairs, and running, which limits patients' ability to engage in daily activity [2]. In addition, recurrent or chronic symptoms are experienced by 70%–90% of patients with initial PFP [3], and prolonged PFP can lead to patellofemoral arthritis [4], significantly impairing individuals' quality of life. Although effective interventions and/or treatments with the goal of preventing PFP and improving patients' outcomes are needed, 60% of clinical interventions yielded non‐significant outcomes in patients who had PFP [5]. Conservative interventions such as patellar taping and bracing, foot orthoses, and muscle strengthening exercises targeting the thigh and hip musculature are commonly used for clinical interventions; however, their efficacy lacks robust evidence [6, 7].

Identifying effective methods to reduce patellofemoral joint stress (PFJS) is crucial for PFP prevention and treatment as elevated PFJS was proposed, as the primary cause of PFP [8]. Previous studies have shown that factors such as foot strike patterns and running shoes can influence PFJS [9, 10, 11]. When habitual rearfoot strikers with PFP transition to forefoot strike, there is a decrease in the patellofemoral joint reaction force and PFJS, resulting in pain relief [9]. Moreover, different designs of running shoes impact the foot strike pattern during running [11, 12, 13, 14]. Consequently, modifying running shoes could potentially reduce patellofemoral joint loading and alleviate pain.

A previous study found that healthy people who typically land on their rearfoot while running tend to transition to a midfoot or forefoot strike pattern when wearing negative heel shoes [13]. Compared with running shoes with a high drop, running in shoes without a drop decreases the foot inclination angle and peak PFJS in healthy individuals [11, 15]. However, there are differences in lower limb biomechanical characteristics between PFP patients and healthy individuals during running [16, 17, 18, 19]. Consequently, it remains unclear whether negative heel running shoes can influence perceived pain and lower limb biomechanical characteristics, such as PFJS, in runners with PFP.

Therefore, the purpose of this study was to determine the effects of negative heel shoes on perceived pain, foot strike pattern, and knee biomechanical characteristics during running in PFP runners, and to investigate the effects of wearing negative heel running shoes on relieving knee pain and its biomechanical mechanisms. The hypotheses were that PFP runners who habitually land on the rearfoot while wearing negative heel shoes would result in: (1) a decrease in perceived pain; (2) a transition to forefoot strike during running; (3) a decrease in the peak PFJS during the stance period; (4) a decrease in knee flexion angle, knee extension moment, quadriceps force, patellofemoral joint reaction force, an increase in quadriceps moment arm, and patellofemoral contact area at the time of peak PFJS.

## 2 METHODS

### 2.1 Participants

Sixteen male runners (age [mean ± standard deviation]: 20.6 ± 2.0 years, body height: 1.76 ± 0.05 m, body mass: 68.89 ± 4.88 kg) with PFP recruited in this study. The PFP was screened by (1) experiencing peripatellar or retro‐patellar pain during running and at least one of the following movements: squatting, ascending/descending stairs, prolonged sitting, hopping, and resisted knee extension; (2) experiencing minimum pain levels of 3/10 on a visual analog scale (VAS) (0 for no pain, 10 for maximal pain); and (3) experiencing pain for more than 3 months without direct trauma as the cause [20]. Additionally, participants included in this study if they (1) are rearfoot strikers (the foot inclination angle of participants running in positive heel shoes was >8° [21]); (2) have no prior experience of running in negative heel shoes (the stack height of the forefoot parts of the sole is greater than that of the heel parts); (3) having a running experience of at least 2 years; and (4) running at least 10 km per week. Participants were excluded if they have had (1) previous patellar subluxation or dislocation; (2) concomitant injury or pathology of other knee structures (e.g., menisci, ligamentous, patellar tendon, iliotibial band); and (3) history of lower limb surgery.

### 2.2 Shoe materials

**FIGURE 1:**
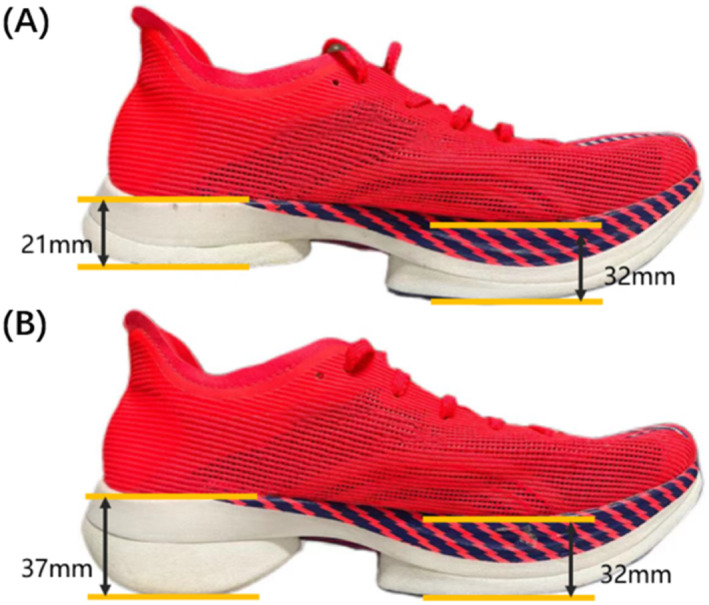
Negative heel‐to‐toe drops (A) and positive heel‐to‐toe drops (B) of the test shoes.

The running shoes were custom‐made by a professional shoe factory with the same forms and structures other than different heel‐to‐toe drops (Figure 1). The two heel‐to‐toe drops include one negative drop (21 mm at heel, 32 mm at toe, for −11 mm drop) and one positive drop (37 mm at heel, 32 mm at toe, for 5 mm drop). The shoe size was 41 EU, the sole material was polyurethane with a density of 0.10 g/cm^3^, and the midsole harness was 42 Asker C.

### 2.3 Procedures

Participants changed into uniform compression tights and performed a standard warm‐up protocol. Each participant underwent a 10‐min warm‐up session involving walking and running to adapt to the test shoes and the target speed before the official test. Nineteen retroreflective markers were placed on participants' left/right anterior superior iliac spine, the midpoint of the fourth and fifth lumbar spine, left/right anterior thigh, left/right lateral femoral condyle, left/right medial femoral condyle, left/right tibial tuberosity, left/right lateral fibular malleolus, left/right medial tibial malleolus, left/right toe, and left/right heel.

For the official trials, participants ran on a 20 m long runway aiming for a speed of 4 ± 0.3 m/s [11, 20] in the focused testing area. A portable speed measurement system (Smartspeed) was placed at both sides of the force plates with a spacing of 3 m to monitor the running speed. No additional interventions were performed during the test, and participants were not given any specific instructions regarding their running pattern or pace adjustment while wearing the test shoes. Three successful trials under each condition were collected. There were two conditions, with participants wearing negative and positive drops in a randomized order. A trial was considered successful if they (1) landed on the force plate (Kistler 9287CA, 1000 Hz) located in the middle of the focused testing area with the affected foot. If participants reported bilateral symptoms, they were required to use the limb subjectively reported as the most painful; (2) the retroreflective markers were captured by a motion capture system (Oqus700, 200 Hz); (3) the running speed over the force plate had to be within the range of 4 ± 0.3 m/s; (4) maintain a consistent pace without making any pace adjustments before landing on the force plate.

After completing the running test for each pair of shoes, the participants used a 10‐cm VAS to assess their perceived pain during running for each condition. The VAS consisted of a horizontal line, 10 cm in length, on which the participants marked their perceived pain level. A VAS score of 0 indicated no pain, while a score of 10 indicated extremely intense pain [9].

### 2.4 Data reduction

The three‐dimensional coordinate data of the markers were filtered by a Butterworth recursive low‐pass digital filter with a cut‐off frequency of 13.3 Hz [22]. The pelvis, thigh, shank, and foot reference systems were established based on the markers' coordinates. The hip joint center was identified based on Bell's research [23], and the knee and ankle joint centers were determined as previously described [24]. Euler angles method and inverse dynamics methods were used to calculate the knee joint angle and moment [25, 26].

Dependent variables were calculated during the stance phase of the running. The stance phase was defined as the initial contact of the affected foot until subsequent toe‐off on the same side. The initial contact and toe‐off were defined as the first frame that the vertical ground reaction force was greater or smaller than 10 N, respectively. The dependent variables included VAS scores, foot inclination angle, knee joint angle and moment, patellofemoral joint reaction force and stress, patellofemoral contact area, quadriceps force, and moment arm. The joint moment data was normalized by the product of the subject's height and body weight, and joint forces and quadriceps forces were normalized by the subject's body weight. Additionally, the dependent variables of the affected limb were analyzed. When participants reported bilateral symptoms (*n* = 10), the limb subjectively reported as the most painful by participants was analyzed.

In this study, we defined the foot inclination angle as the angle between the vector connecting the toe and heel markers at the landing and the anteroposterior axis in the laboratory coordinate system. The strike pattern was determined according to the foot inclination angle, in which foot inclination angle >8° was considered rearfoot landing, −1.6°< foot inclination angle <8° was considered midfoot landing, and foot inclination angle < −1.6° was considered forefoot landing [21].

The patellofemoral joint reaction forces and stresses were calculated according to the model of Whyte [27] and the regression equation of the patellofemoral contact area obtained by Vannatta [28].

### 2.5 Statistical analysis

A paired‐sample *t*‐test was used to determine the effect of heel‐to‐toe drop of running shoes on VAS scores, foot inclination angle, and biomechanical parameters of the knee joint in PFP runners with PFP. A type I error rate no >0.05 was chosen as an indication of statistical significance, and all statistical analyses were performed using SPSS 24.0 (IBM). Effect sizes were calculated using Cohen's *dz*, with Cohen's *dz* < 0.5 being defined as “small,” 0.5 ≤ Cohen's *dz* ≤ 0.8 as “medium,” and Cohen's *dz* > 0.8 as “large.”

## 3 RESULTS

### 3.1 Effects on perceived pain

There was no significant difference in VAS scores between participants running in negative heel shoes (3.2 ± 1.5) and positive running shoes (3.4 ± 1.7) (*p* = 0.517, Cohen's *dz* = 0.17).

### 3.2 Effects on foot inclination angle

The foot inclination angle was smaller in negative heel shoes (16.1° ± 9.1°) compared to positive heel shoes (19.9° ± 5.2°) (*p* = 0.013, Cohen's *dz* = 0.68). Additionally, two participants (12.5%) exhibited a foot inclination angle of < −1.6° when wearing negative heel shoes. If these two subjects were not included, the foot inclination angle was still smaller in negative heel shoes (19.3° ± 4.3°) compared to in positive heel shoes (21.2° ± 3.9°) (*p* = 0.032, Cohen's *dz* = 0.64).

### 3.3 Effects on knee angle and moment in sagittal plane

**FIGURE 2:**
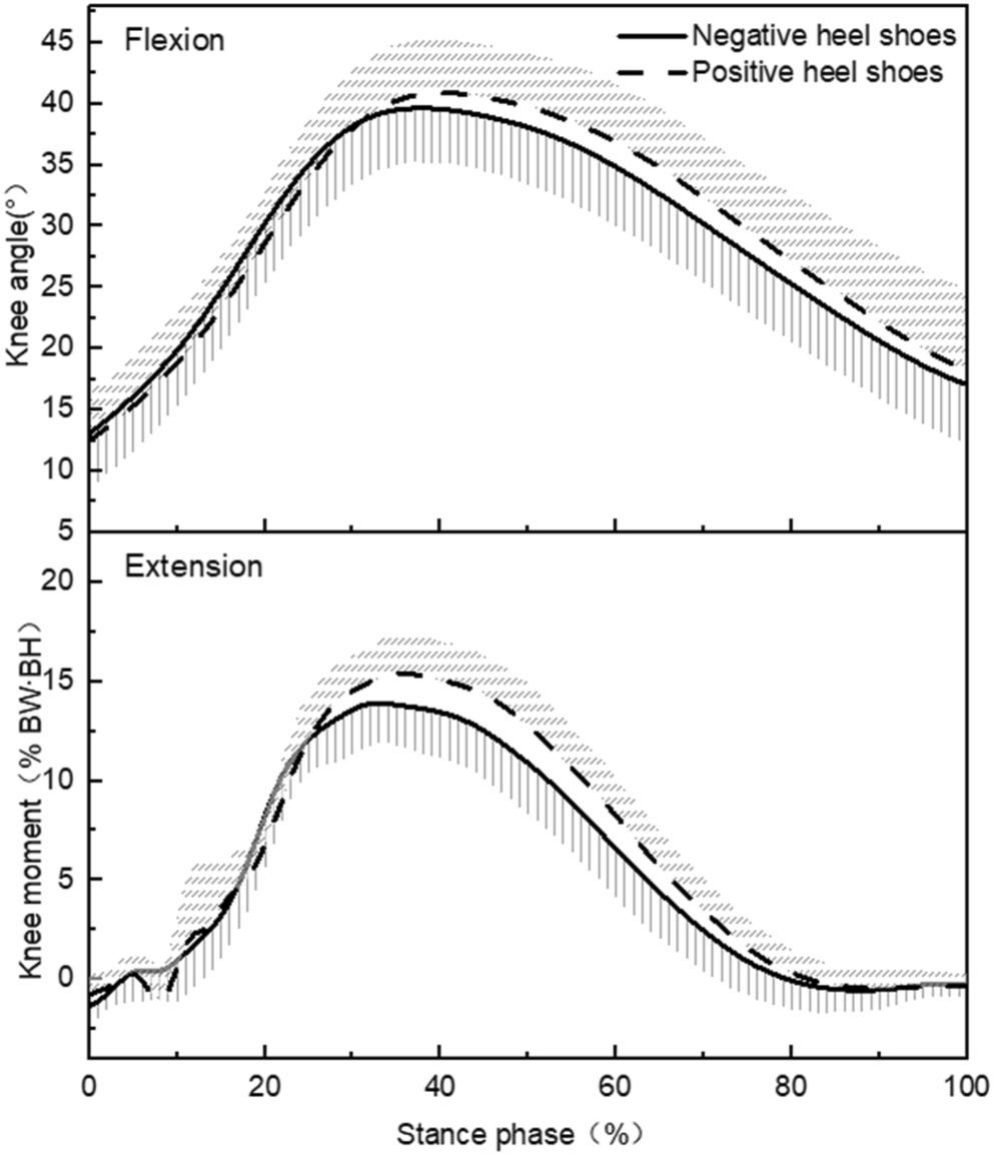
The curves of knee joint angle and moment in shoes with different heel‐to‐toe drops.

The traces of changes in knee angle and moment of the stance phase during running in different heel‐to‐toe drop shoes are shown in Figure 2, and the results of the paired‐samples *t*‐test showed (Table 1) that the knee moment at the time of peak PFJS during the stance phase was lower in negative heel shoes compared to positive heel shoes (*p* = 0.003, Cohen's *dz* = 0.89). However, the knee flexion angle (*p* = 0.354, Cohen's *dz* = 0.24) at the time of peak PFJS did not find a significant difference between negative heel shoes and positive heel shoes.

**TABLE 1:** Dependent variables: comparison of knee joint angle and moment at the time of peak patellofemoral joint stress ($\bar{x}\pm s$).

Variables	Negative heel shoes	Positive heel shoes	*p*	Cohen's *dz*
Knee flexion angle at time of peak PFJS (°)	40.2 ± 4.4	40.7 ± 4.4	0.354	0.24
Knee extension moment at the time of peak PFJS (%BW·BH)	14.18 ± 2.30	15.46 ± 2.39	0.003	0.89

Abbreviations: BH, body height; BW, body weight; PFJS, patellofemoral stress.

### 3.4 Effects on the biomechanical parameters of patellofemoral joint

**FIGURE 3:**
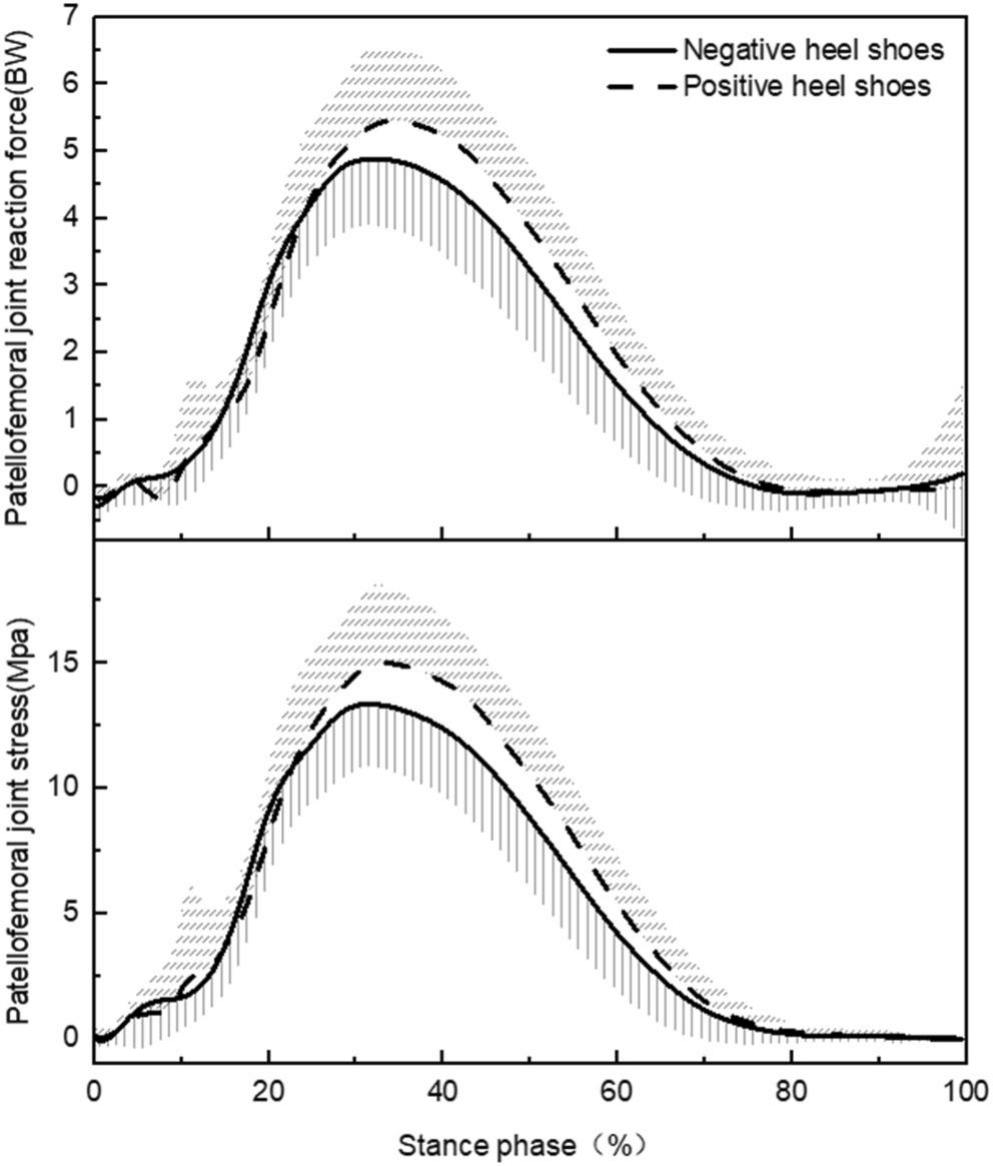
The curves of patellofemoral joint reaction force and stress in shoes with different heel‐to‐toe drops.

The traces of changes in patellofemoral reaction force and stress of the stance phase during running in different heel‐to‐toe drop shoes are shown in Figure 3, and the results of the paired‐samples *t*‐test showed (Table 2) that the peak PFJS (*p* = 0.006, Cohen's *dz* = 0.81) during the stance phase, the patellofemoral joint reaction force (*p* = 0.007, Cohen's *dz* = 0.78) and quadriceps force (*p* = 0.004, Cohen's *dz* = 0.84) at the time of peak PFJS was lower in negative heel shoes compared to positive heel shoes. No significant difference in the patellofemoral contact area (*p* = 0.501, Cohen's *dz* = 0.17) and quadriceps moment arm (*p* = 0.332, Cohen's *dz* = 0.25) at the time of peak PFJS was not found between negative heel shoes and positive heel shoes.

**TABLE 2:** Dependent variables: comparison of biomechanical parameters of patellofemoral joint when running in shoes with different heel‐to‐toe drops ($\bar{x}\pm s$).

Variables	Negative heel shoes	Positive heel shoes	*p*	Cohen's *dz*
Peak PFJS (MPa)	14.44 ± 2.54	15.84 ± 2.94	0.006	0.81
Patellofemoral joint reaction force at the time of peak PFJS (BW)	5.10 ± 1.04	5.60 ± 1.09	0.007	0.78
Patellofemoral contact area at the time of peak PFJS (mm^2^)	236.24 ± 8.24	237.12 ± 7.51	0.501	0.17
Quadriceps force at the time of peak PFJS (BW)	6.13 ± 1.09	6.70 ± 1.14	0.004	0.84
Quadriceps moment arm at time of peak PFJS (cm)	4.15 ± 0.16	4.14 ± 0.16	0.332	0.25

Abbreviations: BW, body weight; PFJS, patellofemoral stress.

## 4 DISCUSSION

Pain is the primary symptom of PFP, and changes in pain level may result in the compensatory strategies observed during functional activities [20]. Reasonable interventions for PFP should serve to alleviate pain [20]. The VAS is a widely utilized method for assessing knee pain in patients with PFP [29]. Our results showed that there was no significant difference in VAS scores between PFP runners running in negative heel and positive heel running shoes, which did not support the hypothesis that perceived pain is reduced when PFP runners wear negative heel shoes. This might be related to the fact that this study only examined the acute effect of negative heel running shoes on the VAS scores of PFP patients while running. Given the limited amount and intensity of the exercise, the acute effects of negative heel running shoes might not have been sufficient to elicit changes in VAS scores during short‐distance running. Similarly, it was observed that knee taping had no immediate impact on VAS scores [30], while extended utilization of patellofemoral taping led to a decrease in VAS scores [31]. Future studies could consider collecting VAS scores from PFP runners after completing long‐distance runs wearing negative heel running shoes to provide a more realistic representation of running exercise.

The results of this study partially supported the research hypothesis that running in negative heel shoes leads to a switch in foot strike pattern towards a forefoot strike. The results showed that the foot inclination angle was 3.8° lower when running in negative heel shoes compared to running in positive shoes. Additionally, two participants displayed a foot inclination angle of < −1.6° when running in negative heel shoes. Yu [13] also found that although not all healthy runners accustomed to rearfoot strike used the forefoot or midfoot strike, the foot strike pattern shifted anteriorly when wearing negative heel shoes. The reason why not all participants experienced a shift in foot strike pattern when directly replacing negative heel running shoes might be related to the individual differences in the adaptive ability of the foot and ankle muscles to negative heel running shoes in patients with PFP. The result revealed that compared with the participants who maintained rearfoot landing in negative heel shoes, the two participants who exhibited a forefoot strike in negative heel shoes demonstrated a lower foot inclination angle in positive heel shoes. This might suggest that the two participants possessed stronger foot and ankle muscle to accommodate the negative heel shoes, primarily relying on an increased ankle plantarflexion angle during landing. Conversely, the other participants who maintained their original strike pattern might rely on a combination of decreased ankle dorsiflexion angle and knee flexion angle during landing. Furthermore, previous studies have shown a positive correlation between the heel‐to‐toe drop of running shoes and foot inclination angle [32, 33]. In this study, the heel‐to‐toe drop of negative heel running shoes was relatively small, and future studies could explore the possibility of further reducing the heel‐to‐toe drop of negative heel running shoes to ascertain whether this modification leads to a forefoot strike pattern.

The results of this study showed that wearing negative heel shoes resulted in a decrease in the foot inclination angle among PFP runners, leading to a shift towards a forefoot strike pattern, however, no significant effect on VAS scores was observed. A previous study showed that PFP runners accustomed to rearfoot strike had significantly lower VAS scores when they altered their foot strike pattern to forefoot strike through gait retraining [9]. Our results showed that both participants who switched their landing patterns experienced reduced VAS scores and perceived pain when running in negative heel shoes. These results suggest that switching to forefoot strike might be more effective in reducing perceived pain among PFP runners. Future studies should investigate the effects of negative heel running shoes with a smaller heel‐to‐toe drop and assess the long‐term impact of wearing such shoes on knee pain in PFP patients to further clarify whether negative heel running shoes can effectively alleviate knee pain in PFP runners.

Repetitive high‐frequency loading of the patellofemoral joint will increase the subchondral bone metabolic activity and the patella water content [34]. The elevated water content may increase the intrapatellar intraosseous pressure, which can stimulate mechanical nociceptors sensitive to pressure and lead to the generation of pain [34]. Most researchers believe that elevated PFJS is the main reason for the occurrence of PFP [8]. This study found that the peak PFJS in PFP runners running in negative heel running shoes was less than that in positive heel running shoes, which supported the third hypothesis.

The increase in PFJS can be attributed to either an increase in patellofemoral joint reaction force or a decrease in contact area [7]. The results of this study demonstrated that PFP runners experienced a reduction of more than 8.8% in patellofemoral joint reaction force when wearing negative heel shoes compared to positive heel shoes, while no significant difference in the patellofemoral joint contact area was detected. Therefore, the decrease in patellofemoral joint contact force was identified as the primary reason for the stress reduction observed in PFP runners wearing negative heel shoes. Patellofemoral joint reaction force is the resultant force resulting from the actions of the quadriceps muscle and patellar tendon on the patella and is positively correlated with the quadriceps force [8]. The results of this study showed that the quadriceps force at the time of peak PFJS was lower when wearing negative heel shoes compared to positive heel shoes, consistent with the findings regarding patellofemoral joint contact force. The decrease in quadriceps force was associated with a decrease in knee extension moment and an increase in its moment arm. Our study found that the knee extension moment at the time of peak PFJS was significantly smaller when wearing negative heel shoes compared to positive heel shoes, while there was no significant difference in the quadriceps moment arm. These results suggest that the decrease in peak PFJS in PFP runners wearing negative heel running shoes can be primarily attributed to the decrease in knee extension moment. When running in negative heel shoes, PFP runners exhibited a reduced foot inclination angle and the foot strike pattern shifted anteriorly, which might lead to the lower‐extension moment [13, 15]. This is consistent with the finding of Xu et al. [35], which indicated that the primary reason for the lower PFJS observed in forefoot strike compared to rearfoot strike was the decrease in knee extension moment.

The relationship between VAS scores and PFJS in patients with PFP remains unclear. Most studies have found that when PFJS is small, VAS scores are correspondingly smaller [9, 36]. VAS scores were also found to be smaller, but there was no significant difference in PFJS in PFP runners running with knee sleeves compared to those running without knee sleeves [37]. Our results showed a significant decrease in peak PFJS with a large effect size when PFP runners wore negative heel shoes compared to positive heel shoes, however, there was no significant difference in the VAS scores. This finding may be attributed to the relatively small decrease in peak PFJS (8.8%) observed when running in negative heel shoes. Additionally, it might be related to the influence of subjective perception or cumulative fatigue on the VAS scores.

There were several limitations of this study. Firstly, the biomechanical model used to calculate the patellofemoral joint load in this study was based on previous cadaveric studies. The input parameters, knee extension moment, and knee flexion angle did not account for individual differences or movements in the horizontal and coronal planes. This discrepancy between the calculated results and real situations could be addressed in future research by utilizing techniques like dual fluoroscopic imaging systems to accurately measure the contact area of the patellofemoral joint. Additionally, a more comprehensive and accurate patellofemoral biomechanical model could be employed to calculate the mechanical parameters, thereby improving the reliability of stress calculations. Secondly, knee pain may have a certain effect on the biomechanical characteristics of the knee joint [20]. In this study, the consistent pain level was not strictly controlled before testing each pair of running shoes. To avoid experimental errors arising from inconsistent pain levels, future studies should ensure strict control over pain levels. Furthermore, the participants in this study had a habitual rearfoot strike pattern. However, it is worth considering that the biomechanical characteristics of the lower limbs differ for different landing styles [35]. Therefore, it is prudent to generalize to PFP patients who are used to forefoot landing. Lastly, it is important to acknowledge that the participants in this study were exclusively male. Biomechanical characteristics can vary among PFP patients of different genders, with women having a higher risk of PFP than men [38]. Therefore, future studies should be expanded to include female PFP patients to provide a more comprehensive understanding of the topic.

## 5 CONCLUSION

Compared to positive heel running shoes, running in negative heel shoes decreased the foot inclination angle of PFP runners, leading to a decrease in the peak PFJS achieved by decreasing the knee extension moment at the time of peak PFJS, which may decrease patellofemoral joint loading, thus reducing the possibility of further development of PFP. Before changing the landing style, habitual rearfoot strikers with PFP could consider changing the negative heel running shoes to reduce the patellofemoral joint load, thus reducing the likelihood of further injury progression.

## AUTHOR CONTRIBUTIONS

**Yu Gu**: Data curation (lead); formal analysis (lead); writing – original draft (lead); writing – review & editing (equal). **Zhiyi Zheng**: Resources (equal). **Quanshou Zeng**: Resources (equal). **Chen Yang**: Writing – review & editing (equal). **Yu Song**: Writing – review & editing (equal). **Xianglin Wan**: Conceptualization (lead); methodology (lead); project administration (lead); writing – review & editing (equal).

## CONFLICT OF INTEREST STATEMENT

The authors declare that they have no competing interests.

## ETHICS STATEMENT

Written informed consent was obtained from all participants prior to testing. The ethical approval was granted by the Sports Science Experiment Ethic Committee of Beijing Sport University (2023095H).

## CONSENT FOR PUBLICATION

Not applicable.

## Data Availability

The databases used and/or analyzed during the current study are available from the corresponding author upon reasonable request.
